# Assessment of Coverage in England of Cancer Drugs Qualifying for US Food and Drug Administration Accelerated Approval

**DOI:** 10.1001/jamainternmed.2020.8441

**Published:** 2021-02-22

**Authors:** Avi Cherla, Huseyin Naci, Aaron S. Kesselheim, Bishal Gyawali, Elias Mossialos

**Affiliations:** 1 Department of Health Policy, London School of Economics and Political Science, London, United Kingdom; 2 Program on Regulation, Therapeutics, and Law, Division of Pharmacoepidemiology and Pharmacoeconomics, Brigham and Women’s Hospital, Harvard Medical School, Boston, Massachusetts; 3 Department of Oncology and Department of Public Health Sciences, Division of Cancer Care and Epidemiology, Queen’s University, Kingston, Canada

## Abstract

**Importance:**

Numerous cancer drugs have received accelerated approval from the US Food and Drug Administration (FDA) based on clinical trial outcomes that are otherwise not acceptable for traditional FDA approval; the accelerated approval process allows outcomes based on surrogate measures that are only reasonably likely to estimate clinical benefits. In England, the National Institute for Health and Care Excellence (NICE) evaluates the clinical benefits and cost-effectiveness of drugs after they have received regulatory approval and issues recommendations regarding their coverage in the National Health Service (NHS). However, the level of concordance between European and FDA decision-making in the context of drugs qualifying for FDA accelerated approval is unknown.

**Objective:**

To compare FDA accelerated approval decisions for cancer drugs with NICE coverage decisions.

**Design, Setting, and Participants:**

This retrospective cohort study compared cancer drug indications that received FDA accelerated approval from December 11, 1992, to May 31, 2017, with the same set of drug indication pairs in England until August 31, 2019. Data from European Public Assessment Reports developed by the European Medicines Agency (EMA) and public appraisal documents from NICE were used to determine NHS coverage recommendations. National Institute for Health and Care Excellence (NICE) public appraisal documents were analyzed for drug indications, characteristics of clinical evidence, cost-effectiveness, and coverage decisions. Data were analyzed from September 1 to December 31, 2019.

**Main Outcomes and Measures:**

Cancer drug indication coverage decision by NICE.

**Results:**

From 1992 to 2017, 93 cancer drug indications received FDA accelerated approval, 6 of which were subsequently withdrawn, leaving 87 cancer drug indications on the market. As of August 2019, 5 of these indications had been withdrawn or denied market authorization for the European Union by the EMA. From the cohort of EMA-approved drugs, an additional 7 drug indications were not recommended by NICE and were not deemed to have sufficient clinical benefits or cost-effectiveness to warrant mandatory public coverage in England; 5 drugs were not recommended based on clinical benefit and cost-effectiveness criteria, and 2 drugs were not recommended based on cost-effectiveness criteria alone. In total, 12 drug indications were not recommended for public coverage in the NHS, and an additional 30 drug indications were not reviewed by either the EMA (14 drug indications) or NICE (16 drug indications) by the study end date. Most drug indications recommended by NICE were conditional on the negotiation of additional confidential discounts, the imposition of restricted indications that limited prescribing to specific patient subgroups, or the collection of additional data. Among the 9 drug indications with evidence of overall survival benefit at the time of NICE review, 2 were not recommended for public funding based on cost-effectiveness criteria.

**Conclusions and Relevance:**

In this cohort study, 30 cancer drug indications that were granted accelerated approval by the FDA were not subsequently reviewed by either European regulators or NICE, and 12 drugs were denied authorization or coverage owing to insufficient safety, clinical efficacy, or cost-effectiveness. National Health Service coverage of cancer drugs given FDA accelerated approval commonly required additional price concessions, restrictions to approved indications, or review of additional data.

## Key Points

**Question:**

Are cancer drugs granted accelerated approval from the US Food and Drug Administration (FDA) recommended for funding through the National Health Service (NHS) in England?

**Findings:**

In this cohort study of 93 cancer drug indication pairs that received accelerated approval from the FDA between 1992 and 2017, 6 drug indications were withdrawn from the US market, and 42 drug indications were not routinely available through the NHS in England; of those not covered by the NHS, 12 drug indications were not recommended by European regulators or the National Institute for Health and Care Excellence (NICE) because they were deemed to have insufficient safety, efficacy, or cost-effectiveness, and 30 drug indications were not reviewed. Among the drug indications recommended by NICE, 86% required the negotiation of additional confidential discounts, the imposition of restricted indications that limited prescribing to specific patient subgroups, or the submission of additional evidence of efficacy.

**Meaning:**

This study found that many cancer drug indications that received accelerated approval from the FDA were either not reviewed or denied authorization or coverage by European regulators and NICE because of insufficient safety, clinical efficacy, or cost-effectiveness, which was likely owing to the use of uncertain evidence derived from unvalidated surrogate measures, which provided the basis for US regulatory approval.

## Teaser

This cohort study assesses the concordance between cancer drug indications receiving accelerated approval from the US Food and Drug Administration between 1992 and 2017 and those subsequently authorized for funding through the National Health Service in England.

## Introduction

The US Food and Drug Administration’s (FDA) accelerated approval process allows drugs to be approved based on clinical trial findings that would otherwise not be acceptable for use in the traditional FDA approval process (ie, indicating changes based on surrogate measures that are only reasonably likely to estimate actual clinical benefit).^1^ Surrogate measures include biomarkers and other measurable physical properties that may be able to estimate the way a patient feels, functions, or survives. When surrogate measures are validated as being clinically meaningful, they can be substituted for traditional clinical outcomes as end points for clinical trials used for FDA approval, allowing those clinical trials to be conducted more quickly or among fewer patients. The accelerated approval process was developed to facilitate the approval of drugs that address unmet medical needs by allowing regulatory approval based on unvalidated surrogate measures; this process requires that the manufacturer commit to conducting confirmatory clinical trials after approval is granted. Accelerated approval is thus comparable to conditional approval; however, the drugs are formally designated as fully approved from the time of their first approval.

Cancer drugs comprise the largest category of drugs that are granted accelerated approval. From 1992 to 2019, more than one-half of drug indication pairs with FDA accelerated approval have been for the treatment of cancer.^1^ In 2018, 83% of drugs approved via this process were indicated for the treatment of solid tumors and hematological cancers.^1^ Previous studies of cancer drugs undergoing accelerated approval indicated that most of these drugs are eventually granted traditional approval.^2,3^ However, confirmatory clinical trials are also likely to use surrogate measures of tumor response or disease progression and, in some cases, to use the same surrogate measures as those used to support the original accelerated approval decision.^2,4^ Surrogate measures of disease response are controversial in the context of cancer drugs,^5^ as some widely used surrogate measures (ie, response rate and progression-free survival) do not have a clear association with improvements in clinically meaningful outcomes, such as overall survival^6^ or quality of life.^7^

The FDA accelerated approval program has implications for other health care systems, as new drugs often enter the US market first.^8^ In Europe, decisions from the European Medicines Agency (EMA) closely align with those of the FDA, and the EMA typically relies on the same set of clinical trial evidence.^9^ Once approved by European regulators, national-level health technology assessment organizations use transparent criteria to evaluate the clinical benefits and cost-effectiveness of new cancer drugs. In England, the National Institute for Health and Care Excellence (NICE) is responsible for informing the funding decisions of the National Health Service (NHS). A comparable assessment process does not exist within the US government.

The concordance between European and US decision-making in the context of drugs granted FDA accelerated approval is unknown. The ways in which NICE subsequently evaluates drugs that are granted accelerated approval by the FDA can provide insight on the frequency with which drugs offering reasonable clinical benefits and cost-effectiveness emerge from the accelerated approval process and can inform future regulatory policy for cancer drugs in both settings. We therefore reviewed coverage decisions by NICE for all new cancer drugs that were granted FDA accelerated approval over the last 25 years. Our hypothesis was that fewer drugs receiving FDA accelerated approval on the basis of uncertain evidence would be recommended for public NHS funding owing to the routine use of comparative clinical benefit and cost-effectiveness assessments by NICE.

## Methods

### Sample Identification and Data Extraction

We evaluated cancer drugs approved through the FDA accelerated approval process from the introduction of the program on December 11, 1992, until May 31, 2017. Drugs were identified from a review of cancer drugs receiving accelerated approval published by the FDA in 2018.^3^ Data on cancer drug indications that were approved using surrogate measures through the accelerated approval program were then compared with information about the same set of drug indication pairs in England. The matching process for drug indication pairs approved by the FDA was performed using the original accelerated approval indication and the first recommendation from NICE on the same drug indication pair.

In England, drugs must pass 2 levels of evaluation before routine use in the health care system. First, only drugs that receive approval from the EMA can be marketed across member states of the European Union (EU), which included England through 2019. Second, in England, drugs are subsequently evaluated by NICE for use in the NHS. As of 2016, cancer drugs are evaluated by NICE within 90 days of market authorization from the EMA. The NHS must subsequently make the treatment available within 90 days of the published appraisal from NICE, which is financed through the government-funded health care system.

We used data from European Public Assessment Reports produced by the EMA^10^ and appraisal documents from NICE to determine the coverage recommendations for cancer drugs in the cohort.^11^ The European Public Assessment Reports and technology appraisals include detailed information about the assessment of each new drug application submitted by manufacturers. Reports are publicly available and published online, regardless of a positive or negative decision from the EMA and NICE.

### Data Analysis

Evaluations published by the EMA and NICE were analyzed for drug indication, appraisal date, comparator used in clinical trials, primary and secondary end points (overall survival or surrogate), cost-effectiveness (available for NICE data), and recommendations for the EU and the NHS, respectively.

Not all drugs need to be recommended by NICE to be available in the NHS. However, drugs without a positive recommendation from NICE do not have a funding mandate; therefore, they are not routinely available in the NHS. Their availability is subject to funding decisions from the relevant commissioning authority (either NHS England [the public entity that oversees the NHS] or the local clinical commissioning groups, which are responsible for planning and commissioning medical services within a geographic area).^11^ Since the 2016 reform of the Cancer Drugs Fund in England, all new cancer drug indications and drugs are appraised by NICE. We categorized drug indication pairs that did not have a public technology appraisal as being not routinely available in the NHS.

We collected information about cancer drugs that were approved with financial and other managed entry agreements negotiated between the NHS, NICE, and the manufacturer. Drugs that were withdrawn or denied market authorization by the EMA and simultaneously reviewed by NICE were noted. Information was current as of the study end date.

## Results

**Figure 1.**
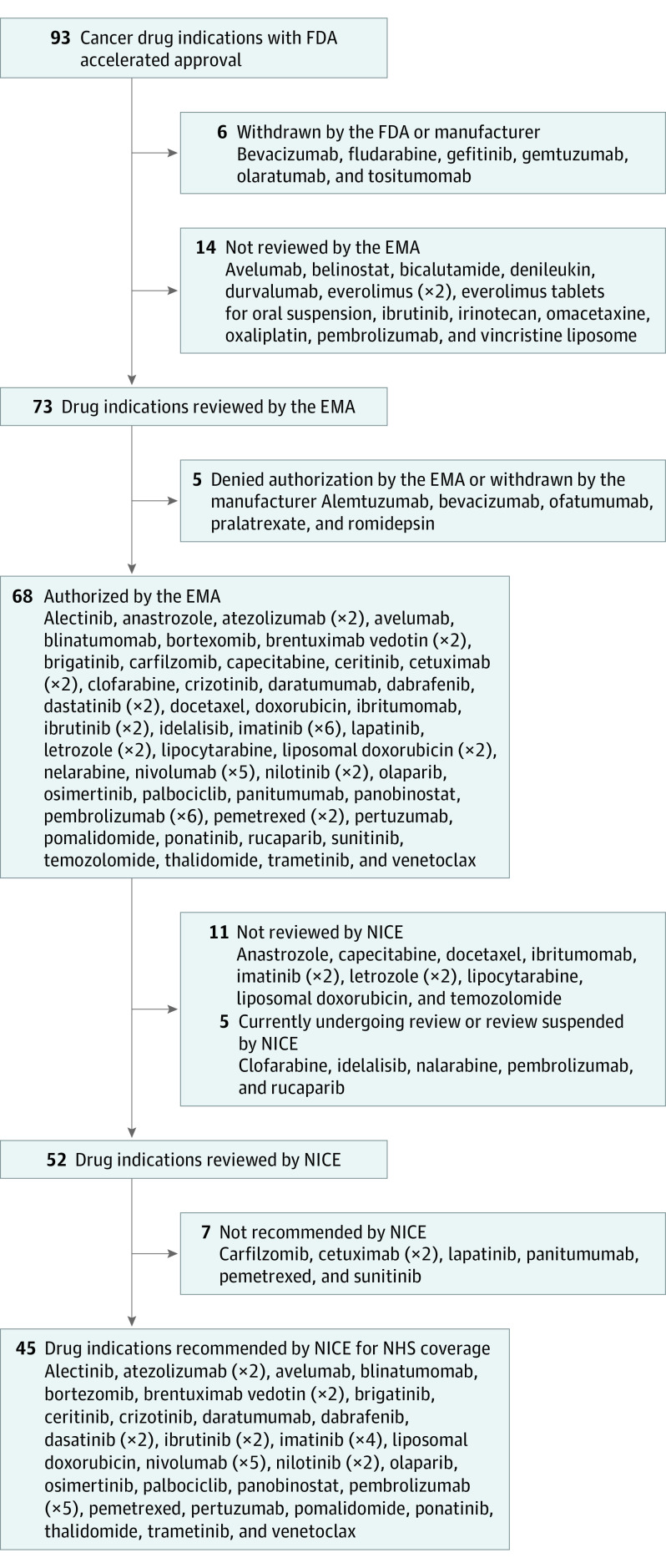
Flow Diagram of Cancer Drug Indications With FDA Accelerated Approval, 1992-2017 Parentheses containing x2, x4, x5, and x6 indicate that the drug was assessed for 2, 4, 5, or 6 multiple indications, respectively (additional information is available in eTable 1 in the Supplement). EMA indicates European Medicines Agency; FDA, US Food and Drug Administration; NHS, National Health Service; and NICE, National Institute for Health and Care Excellence.

Ninety-three cancer drug indications received FDA accelerated approval during the 25-year period analyzed. Six indications were subsequently withdrawn from the US market by either the FDA or the manufacturer, leaving 87 drug indications on the market. To be conservative in our analysis, we excluded these 6 drugs from our final cohort (Figure 1; eTable 1 in the Supplement).

### EMA Appraisal

Among 87 drug indications with FDA accelerated approval, 7 drugs were approved by the EMA for different indications than those approved by FDA, and 7 drugs were not reviewed by the EMA. Of the 7 drugs that were not reviewed by the EMA for different indications, 5 drugs (71.4%) were granted special rare disease designation and were not formally under review by the EMA by the end of our data collection period, and 2 drugs (28.6%) were nationally authorized for use in EU countries, for which manufacturers bypassed the centralized review (Figure 1). Of the 73 drugs for which similar indications were reviewed by the EMA, 5 drugs (6.8%) were negatively assessed, 3 drugs (4.1%; bevacizumab for glioblastoma, romidepsin, and pralatrexate) were denied EU market authorization because of concerns about safety (severe adverse effects) or clinical effectiveness, and 2 drugs (2.7%; alemtuzumab and ofatumumab) were withdrawn by manufacturers for commercial reasons. Effectiveness concerns included no improvement in overall survival, limited certainty regarding the benefit of a surrogate measure, clinical trial method, and study design (Table 1).

**Table 1.** Overview of Assessments for Cancer Drugs Denied Authorization or Coverage by the FDA, EMA, or NICE^a^

Drug	Indication	FDA	EMA	NICE	Reason
Alemtuzumab	Chronic lymphocytic leukemia	Authorized	Withdrawn	Not reviewed	EMA: withdrawn by the manufacturer (Genzyme) for commercial reasons
Bevacizumab	Breast	Withdrawn	Authorized	Not recommended	NICE: small gain in PFS, no improvement in QOL; cost per QALY of >£82 000
Bevacizumab	Glioblastoma	Authorized	Denied market authorization	In development: suspended	EMA: no improvement in OS; improvement in PFS with limited certainty owing to limitations in clinical trial methodology
Cetuximab	Metastatic colorectal cancer	Authorized	Authorized	Not recommended	NICE: incremental improvement in RR; OS (secondary end point) not significant and not compared with standard of care; cost per QALY of £77 000-£370 000
Fludarabine	Chronic lymphocytic leukemia	Withdrawn	Not reviewed	Not reviewed	EMA: bypassed centralized review procedure from EMA; nationally authorized in Belgium
Gefitinib	Non–small cell lung cancer	Withdrawn	Withdrawn	Not recommended	EMA: withdrawn by manufacturer because OS did not meet requirements of EMA Committee for Medicinal Products for Human Use
Gemtuzumab	Acute myeloid leukemia	Withdrawn	Denied market authorization	Not reviewed	EMA: did not indicate benefit and was insufficient to validate effectiveness without a comparator; few patients achieved complete remission; severe adverse effects
Lapatinib	Breast	Authorized	Authorized	Not recommended	NICE: improved PFS; OS benefit (secondary end point) small and uncertain; no difference in QOL; greater chance of adverse events; cost per QALY of £74 000
Ofatumumab	Chronic lymphocytic leukemia	Authorized	Withdrawn	Withdrawn	EMA: withdrawn by manufacturer (Novartis) for commercial reasons
Olaratumab	Sarcoma	Withdrawn	Withdrawn	Withdrawn	EMA: ANNOUNCE clinical trial indicated no improvement in OS or PFS compared with doxorubicin alone
Panitumumab	Metastatic colorectal cancer	Authorized	Authorized	Not recommended	NICE: PFS benefit of 5 wk compared with best supportive care; no statistically significant improvement in OS; cost per QALY of £110 000-£150 000
Pemetrexed	Non–small cell lung cancer	Authorized	Authorized	Not recommended	NICE: no improvement in OS; noninferiority test did not exclude possibility of marginal loss of efficacy compared with docetaxel; not cost-effective compared with docetaxel (>£1 million per QALY) or best supportive care (>£50 000 per QALY)
Pralatrexate	Peripheral T-cell lymphoma	Authorized	Denied market authorization	In development: suspended	EMA: lacking evidence for efficacy of OS or PFS (tumor response is not a clinical benefit end point and cannot be considered as a surrogate)
Romidepsin	Peripheral T-cell lymphoma	Authorized	Denied market authorization	In development: suspended	EMA: study did not include a comparator and was not possible to assess OS or PFS; manufacturer did not provide certificate of good manufacturing practice
Sunitinib	Renal cell carcinoma	Authorized	Authorized	Not recommended	NICE: improvement in PFS but absence of robust data; cost per QALY of £72 000 and £105 000
Tositumomab	Lymphoma	Withdrawn	Not reviewed	Not reviewed	NA

Abbreviations: ANNOUNCE, A Study of Doxorubicin Plus Olaratumab in Participants With Advanced or Metastatic Soft Tissue Sarcoma; EMA, European Medicines Agency; FDA, US Food and Drug Administration; NICE, National Institute for Health and Care Excellence; NA, not applicable; OS, overall survival; PFS, progression-free survival; QALY, quality-adjusted life-years; QOL, quality of life; RR, response rate.

^a^ Regulatory status for the EMA and NICE is based on the first appraisal. Additional information is available in eTable 2 in the Supplement.

In total, 14 of 87 drug indications (16.1%) with FDA accelerated approval were not reviewed by the EMA for use in the EU, 68 drugs (78.2%) were authorized by the EMA, and 5 drugs (5.7%) were withdrawn or denied market authorization. Eight of the authorized drugs (11.8%) received conditional marketing authorization, which required manufacturers to conduct postmarketing studies.^12^ Overall, among similarly evaluated drugs, we found high concordance (93.2%) in approval decisions between the FDA and the EMA.

### NICE Appraisal

Among 68 cancer drug indications with accelerated approval in the US that also received market approval in the EU, 11 drugs (16.2%) were not submitted for NICE evaluation, and 5 drugs (7.4%) were still under review by the end of our data collection period. Among the 52 drug indications evaluated by NICE, 45 drugs (86.5%) were recommended, and 7 drugs (13.5%) were not recommended for routine use in the NHS as of August 31, 2019. A total of 27 of 45 approved drug indications (60.0%) relied on the same surrogate measures used by the FDA for accelerated approval. In total, 16 of 68 drug indications (23.5%) with accelerated approval were not reviewed by NICE by the end of our data collection period, 45 drug indications (66.2%) were recommended for public coverage through the NHS, and 7 drug indications (10.3%) were not recommended.

Of the nonrecommended group of 7 drug indications, 5 drugs (71.4%) were denied authorization or coverage owing to a combination of clinical benefit and cost-effectiveness concerns. These concerns included immature data or results indicating no improvement in overall survival, minimal improvement in a surrogate measure, marginal benefit vs the standard of care, and overall uncertainty in the evidence base (Table 1; eTable 2 in the Supplement). The remaining 2 drugs (28.6%) were denied coverage by NICE primarily based on low cost-effectiveness. The 7 drugs that were not recommended had a mean cost per quality-adjusted life-year (QALY) of $221 000, which was 3.7 times greater than the mean cost per QALY of drugs in the recommended group (approximately $60 000) and exceeded the cost-effectiveness threshold used by NICE ($26 000-$40 000 per QALY in 2019 US dollars) (Table 2).

**Table 2.** Overview of Assessments for Cancer Drugs Denied Coverage by NICE Based on Cost-effectiveness Criteria

Drug	Indication	FDA	EMA	NICE
Carfilzomib	Multiple myeloma	Authorized. Approved based on RR; confirmatory clinical trial verified PFS benefit; converted to traditional approval (Jan 2016)	Authorized. Statistically significant improvement in PFS; preplanned OS analysis was statistically significant	Not recommended. Lack of cost-effectiveness; OS were immature, PFS gain of 8.7 mo; uncertainty with proportional hazard and parametric distribution used to extrapolate OS in the economic model; cost per QALY likely to be substantially >£41 429
Cetuximab	Metastatic colorectal cancer	Authorized. Approved based on RR; confirmatory clinical trial verified OS benefit; converted to traditional approval (Oct 2017)	Authorized. Immature improvement in OS, PFS, and overall RR	Not recommended. Lack of cost-effectiveness; statistically significant improvement in OS; cost per QALY of £90 000

Abbreviations: EMA, European Medicines Agency; FDA, US Food and Drug Administration; NICE, National Institute for Health and Care Excellence; OS, overall survival; PFS, progression-free survival; QALY, quality-adjusted life-year; RR, response rate.

### Clinical End Points and Surrogate Measures

Among 87 cancer drug indications with FDA accelerated approval that were still on the market, postapproval confirmatory clinical trials indicated improvement in overall survival for 19 drug indication pairs.^2^ At the time of EMA appraisal of the same cancer drug indications, 5 drugs had indicated an overall survival benefit in FDA-mandated postapproval studies. After EMA market authorization but before NICE review, overall survival data matured for an additional 4 drug indications. Therefore, of the drug indications in the cohort reviewed by the EMA and NICE, 43 of 52 drugs (82.7%) lacked overall survival data at the time of review, although 9 drugs (17.3%) subsequently indicated improvements in overall survival (Table 3).

**Table 3.** Cancer Drugs With Verified Benefit for Overall Survival at the Time of Regulatory Approval^a^

Drug	FDA indication	Verified overall survival		
		EMA^b^	NICE^b^	FDA postapproval^b^
Blinatumomab	Philadelphia chromosome–negative relapsed or refractory B-cell precursor acute lymphoblastic leukemia	No	Yes	Yes^c^
Bortezomib	Multiple myeloma after receipt of ≥2 previous therapies	No	Yes	Yes
Capecitabine	Metastatic breast cancer that is refractory to paclitaxel and to an anthracycline-containing regimen	No	No	Yes
Carfilzomib	Multiple myeloma after receipt of ≥2 previous therapies, including bortezomib and an immunomodulatory agent	Yes	No; not recommended	PFS
Cetuximab	Single agent for EGFR-positive metastatic CRC intolerant to irinotecan-based chemotherapy	No	Yes; not recommended	Yes
Dabrafenib	In combination with trametinib for the treatment of patients with unresectable or metastatic melanoma with *BRAF* V600E or *BRAF* V600K variants	No	No	Yes
Docetaxel	Advanced or metastatic breast cancer after receipt of previous chemotherapy	Yes	Not assessed	Yes
Ibrutinib	Treatment of chronic lymphocytic leukemia after receipt of 1 previous therapy	No	No	Yes
Imatinib	Adjuvant treatment after complete gross resection of *KIT* CD117–positive GIST	No	No	Yes
Irinotecan	Metastatic colon or rectal cancer that progressed during receipt of fluorouracil–based therapy	No	No	Yes
Nivolumab	In combination with ipilimumab for *BRAF* wild-type metastatic melanoma	No	No	Yes^c^
Oxaliplatin	In combination with fluorouracil and leucovorin for metastatic CRC that recurred or progressed during receipt of fluorouracil and leucovorin plus irinotecan	No	No	Yes
Panitumumab	EGFR-expressing metastatic CRC during receipt of fluoropyrimidine, oxaliplatin, and irinotecan-containing regimens	No	No; not recommended	Yes
Pembrolizumab	PD-L1–positive metastatic NSCLC during receipt of platinum-containing chemotherapy	Yes	Yes	Yes
Pembrolizumab	Squamous cell carcinoma of the head and neck on or after progression and receipt of platinum-containing chemotherapy	Yes	Terminated appraisal	Yes^c^
Pembrolizumab	First-line treatment in patients with nonsquamous NSCLC in combination with pemetrexed and carboplatin	No	No	Yes^c^
Pemetrexed	Locally advanced or metastatic NSCLC during receipt of chemotherapy	No	No; not recommended	Yes
Pemetrexed	Locally advanced or metastatic NSCLC during receipt of cisplatin	No	Yes	Yes
Temozolomide	Refractory anaplastic astrocytoma after progression and during receipt of regimen containing nitrosourea and procarbazine	No	No	Yes
Thalidomide	Newly diagnosed multiple myeloma	Yes	Yes	Safety
Trametinib	In combination with dabrafenib for the treatment of patients with unresectable or metastatic melanoma with *BRAF* V600E or *BRAF* V600K variants	No	No	Yes

Abbreviations: *BRAF*, B-RAF protooncogene, serine/threonine kinase; EGFR, estimated glomerular filtration rate; EMA, European Medicines Agency; FDA, US Food and Drug Administration; GIST, gastrointestinal stromal tumors; *KIT* CD117, KIT protooncogene, receptor tyrosine kinase, cluster of differentiation 117; CRC, colorectal cancer; NICE, National Institute for Health and Care Excellence; NSCLC, non–small cell lung cancer; PD-L1, programmed cell death 1 ligand 1; PFS, progression-free survival.

^a^ All drug indication pairs were approved by the FDA and the EMA. Four drugs were not recommended by NICE (2 drugs [panitumumab and pemetrexed] because of clinical and cost-effectiveness and 2 drugs [carfilzomib and cetuximab] because of cost-effectiveness alone). In total, 21 cancer drug indications with accelerated approval verified overall survival, from which 19 studies evaluated overall survival in FDA-mandated postmarket confirmatory clinical trials. Docetaxel did not have a public assessment report and was not assessed by NICE (categorized as not routinely available in England). One appraisal from NICE for pembrolizumab was terminated, as evidence was not submitted by the manufacturer. The FDA postmarket confirmatory clinical trial for carfilzomib measured progression-free survival and the confirmatory clinical trial for thalidomide measured safety.

^b^ Indicates the drug had a verified benefit for overall survival at the time of approval by the regulatory agency in the column.

^c^ Gyawali et al^2^ reported that 4 drug indication pairs indicated statistically significant overall survival in postmarket confirmatory clinical trials.

**Figure 2.**
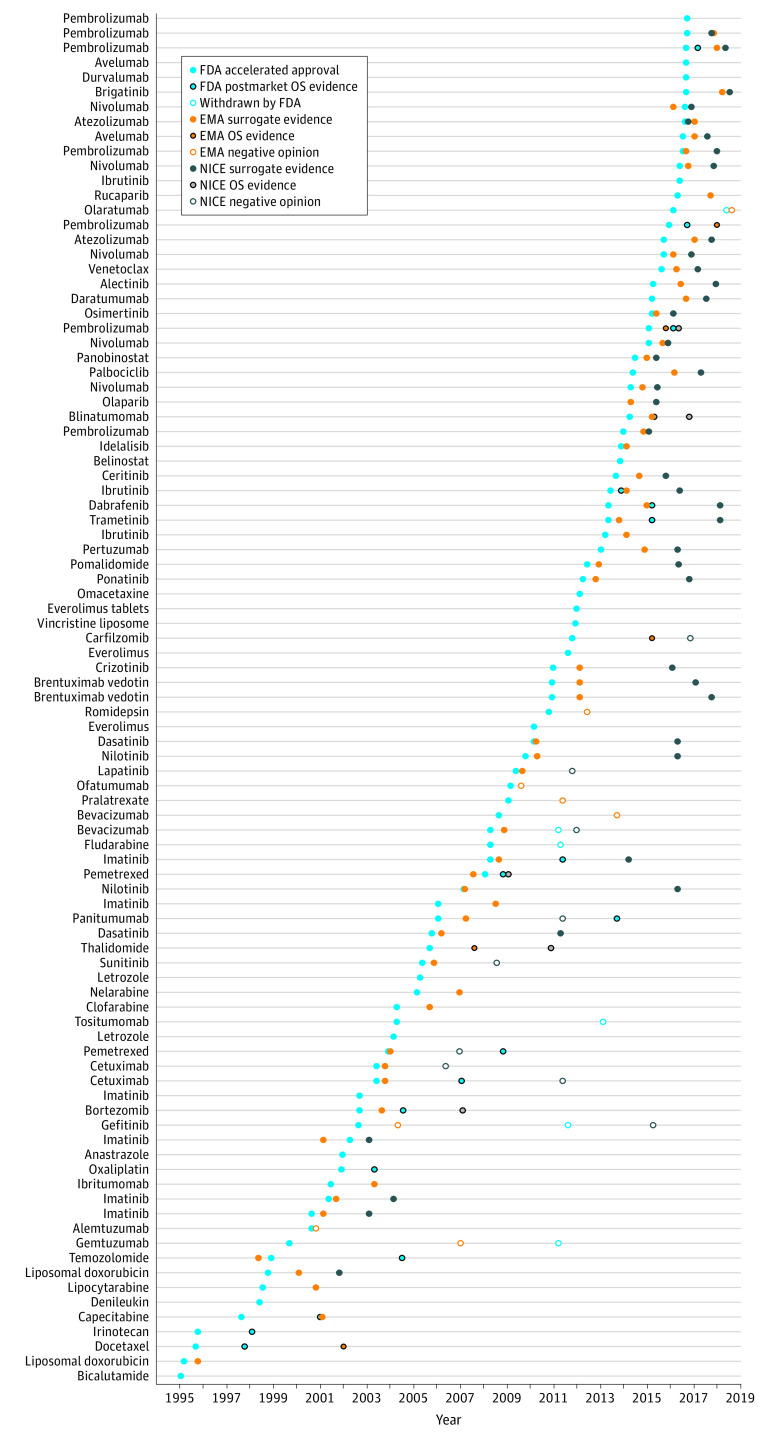
Timeline of Evidence for Accelerated Approval Cancer Drug Appraisals From the FDA, EMA, and NICE The timeline indicates when each drug indication pair was granted FDA accelerated approval and whether subsequent data from postmarket confirmatory clinical trials verified benefits for overall survival. In addition, data from the EMA and NICE were overlaid to present information regarding when these drugs were similarly reviewed in England and whether they were approved based on a surrogate measure (ie, response rate or progression-free survival), whether they had evidence for overall survival, or whether they were denied authorization at the regulatory level by the EMA or not recommended by NICE for NHS coverage because they did not meet the criteria for clinical benefit or cost-effectiveness. The dates on which the FDA verified evidence of overall survival for 4 drug indication pairs from a 2019 review^2^ were listed using the primary completion dates recorded in ClinicalTrials.gov. One indication for imatinib (pediatric Ph-positive chronic-phase chronic myeloid leukemia resistant to interferon or recurrent after receipt of stem cell transplant) evaluated by the EMA based on a surrogate measure did not have a discernable approval date and was not included in the timeline. Anastrozole and 2 indications for letrozole were approved by the EMA but only had a public opinion for harmonizing European Union prescribing; therefore, the date of approval and the clinical end point were unavailable. EMA indicates European Medicines Agency; FDA, US Food and Drug Administration; NHS, National Health Service; NICE, National Institute for Health and Care Excellence; and OS, overall survival.

Of the 9 drug indications with overall survival benefit at the time of EMA and NICE review, 2 drugs (22.2%) were not recommended by NICE for routine coverage because of insufficient cost-effectiveness. The preplanned analysis of the first drug indication, carfilzomib for the treatment of multiple myeloma after the receipt of at least 2 previous therapies (including bortezomib and an immunomodulatory agent), indicated a median overall survival of 48.3 months vs 40.4 months (hazard ratio [HR], 0.79; 95% CI, 0.67-0.95; 1-sided *P* = .005). The second drug indication, cetuximab for the treatment of estimated glomerular filtration rate–positive metastatic colorectal cancer resistant to irinotecan-based chemotherapy, had a median overall survival of 6.1 months vs 4.6 months (HR, 0.77; 95% CI, 0.64-0.92; *P* = .005). Both drug indications exceeded the £20 000-£30 000 per QALY cost-effectiveness threshold used by NICE, with an incremental cost-effectiveness ratio greater than £41 000 ($50 000) per QALY for carfilzomib and £90 000 ($109 000) per QALY for cetuximab. Among the cancer drugs covered by NICE that indicated improvements in overall survival, the median increase was 3.7 months. The timeline of FDA accelerated approval, EMA market authorization, and NICE coverage recommendations according to the availability of evidence on overall survival benefit are shown in Figure 2. The indications for the drugs depicted in the timeline are listed in eTable 3 in the Supplement.

### Cancer Drug Payment in England

Most cancer drug indications (39 of 45 drugs [86.7%]) recommended by NICE required either negotiation between the manufacturer and the NHS for special financial agreements with confidential discounts to improve cost-effectiveness or collection of additional data to verify clinical benefit. A total of 19 of 39 recommended drugs (48.7%) that received agreements required more than 1 agreement designed to improve cost-effectiveness, restrict the drug indication to specific subgroups of patients, or collect additional clinical evidence. Nine of 39 drugs (23.1%) with an agreement were required through the Cancer Drugs Fund to collect effectiveness data in the postmarketing period through a special access program that enables the investigational use of new cancer drugs while additional evidence is being collected to improve the certainty of clinical benefit.

## Discussion

We found discordance between the US and England in the evaluation of cancer drugs that were granted accelerated approval by the FDA; this discordance was likely owing to the reliance on uncertain evidence associated with the use of unvalidated surrogate measures as the basis for US regulatory approval. In a cohort of 93 cancer drug indications that received FDA accelerated approval over the past 25 years, 30 drug indications were not reviewed for coverage in the NHS, and 12 drug indications were denied authorization or coverage by either European regulators or NICE because of insufficient safety, clinical efficacy, or cost-effectiveness. Most recommendations for drug indication pairs in England were conditional on the negotiation of additional confidential price discounts or the collection of postmarket evidence for efficacy.

The FDA accelerated approval process has advantages and limitations. Although it allows faster regulatory approval of qualifying cancer drugs, the association between many surrogate measures and overall survival may be low or unclear^6^; a 2019 review reported that only 20% of cancer drugs with FDA accelerated approval based on surrogate measures confirmed benefits for overall survival in postmarket randomized clinical trials.^2^ Our study found that most cancer drug indications that received accelerated approval from the FDA were also approved by the EMA and NICE based on surrogate measures. Evidence for overall survival was more likely to be available by the time the EMA and NICE reviewed drugs that received FDA accelerated approval, but this evidence was still only available for 9 drug indication pairs licensed in Europe at the time of NICE review. National Institute for Health and Care Excellence decisions commonly relied on the same set of surrogate measures used by the FDA for accelerated approval. Policy makers in England who are currently seeking to expedite NICE appraisals may wish to consider that decreasing review times will further reduce the level of evidence on which NICE decision-making relies.^13,14^

The FDA accelerated approval process requires manufacturers of new cancer drugs that are approved based on surrogate measures to conduct postmarket studies to confirm clinical benefit and maintain marketing authorization. Some of those confirmatory clinical trials have been delayed,^15^ while many of those that have been completed have not reported a clinically meaningful benefit for a patient-centered outcome, such as overall survival.^4^ In England, public funding is not conditional on the completion of postmarket studies for most new cancer drugs that are approved based on surrogate measures. Only a small fraction of drug indications that received FDA accelerated approval and were subsequently recommended for use in the NHS were subject to postmarket evidence collection, and this evidence was usually obtained through observational studies.

Despite uncertainty about the clinical benefit of new cancer drugs receiving accelerated approval, many FDA-approved drugs are routinely covered by public insurance in the US health care system. Medicare Part D and Medicaid state insurance plans are legally required to include almost all FDA-approved drugs in their formularies. In contrast, US private health insurance plans have more flexibility to create limited formularies.^16,17^ Unlike US public insurance programs, which are mandated to cover almost all FDA-approved drugs, we found that the NHS does not routinely cover many cancer drug indications that have received FDA accelerated approval, several of which have had limited evidence of clinical benefit at the time of US regulatory approval. Therefore, England’s government-funded NHS creates a national formulary based on a transparent review of the evidence of clinical benefit and cost-effectiveness, only funding drugs that NICE has found to have the best value.

Among 19 cancer drugs that indicated overall survival benefit in postmarket confirmatory clinical trials, most of the drugs were routinely available through the NHS; however, 4 drugs were not. Two drugs were denied coverage based on insufficient cost-effectiveness, and 2 drugs were not reviewed (Table 3). The appraisal for pembrolizumab for the treatment of squamous cell carcinoma of the head and neck on or after progression during the receipt of platinum-containing chemotherapy was terminated because information was not submitted by the manufacturer. Docetaxel for the treatment of advanced or metastatic breast cancer after previous chemotherapy was not reviewed. Drugs that were not reviewed by NICE could still be made available in the NHS through a commissioning policy or an individual funding request (eg, the clinician believes that a patient’s clinical characteristics are different than those of other patients with the same condition and that the patient would likely respond to treatment). Patients can also access drugs that are not covered in the NHS through the private health care sector in England.

Our finding that several drug indication pairs with FDA accelerated approval were not reviewed by NICE warrants further comment. One explanation is that manufacturers may have recognized that they would not be able to meet the standards imposed by the additional NICE review. Until 2016, when NICE started to review all cancer drug indications approved by European regulators, the prospect of value-based review and evidence-based comparative effectiveness assessments may have deterred manufacturers from submitting drugs for public coverage in the NHS without robust evidence of clinical efficacy or cost-effectiveness, thereby reducing the coverage of drugs with unproven therapeutic benefits and low value for money.

Although we observed high concordance (93.2%) in cancer drug approval decisions between the FDA and the EMA, decision-makers in the US and England have reached different conclusions regarding the safety, efficacy, and cost-effectiveness for a number of cancer drug indications that were granted FDA accelerated approval. Discordance in decisions suggests that relying on unvalidated surrogate measures can create challenges in interpreting evidence, especially in the absence of clinical outcome data. With regard to funding these drugs, there are lessons that public insurance programs in the US can learn from the NHS. First, after clinical outcome data from postmarket confirmatory clinical trials become available, these data can be used to create formularies that are similar to those of the NHS, reevaluating coverage decisions for drugs based on their safety and efficacy benefits. Second, for drugs which have yet to complete confirmatory studies, public insurance programs can make access conditional on price discounts or additional collection of data measuring clinical outcomes, such as overall survival and quality of life.^17^

### Limitations

This study has several limitations. Our cohort of cancer drugs is not representative of all cancer drugs because it is limited to cancer drugs that were first approved in the US through the FDA accelerated approval process, and we did not compare our results with the ways in which cancer drugs that initially receive traditional FDA approval are reviewed by NICE. In a recent European study, drugs included in the EMA’s conditional marketing authorization process were equally likely to be recommended by NICE but were more than 4 times more likely to be conditional on the negotiation of additional discounts or the imposition of restricted indications that limited prescribing to specific patient subgroups.^18^ These results suggest that the proportion of cancer drugs with positive NICE recommendations may not differ among those that received FDA accelerated approval and those that received traditional approval.

Second, information was limited to publicly available drug evaluations based on EMA and NICE assessments of evidence. Recently approved drugs from the FDA will have had less time to be evaluated by NICE, as some assessments are still being conducted. Third, the FDA accelerated approval process was originally established in 1992, while NICE was created in 1999 and first began publishing appraisals in 2000. Therefore, few of the nonreviewed drug indication pairs may have preceded the formation of NICE. Fourth, although surrogate measures used in accelerated approval were, by definition, not validated, we did not assess whether these surrogate measures were later validated, although formal surrogate validation studies are uncommon.^19^

## Conclusions

Among 93 cancer drug indications that received accelerated approval from the FDA, 30 drug indications were not subsequently reviewed by either European regulators or NICE, and 12 drug indications were denied authorization or coverage because of insufficient safety, clinical efficacy, or cost-effectiveness. National Health Service coverage of accelerated approval drugs was often conditional on the negotiation of additional price concessions, the collection of additional data, or the restriction of drug indications to specific patient subgroups. The discordance between the US and England in the evaluation of cancer drugs granted FDA accelerated approval is likely owing to the routine use of comparative clinical and cost-effectiveness assessments by NICE for coverage decisions.

## Notes

Supplement. **eTable 1.** Cancer Drug Indications With FDA Accelerated Approval Not Recommended or Not Reviewed by EMA or NICE **eTable 2.** Overview of Assessments for Cancer Drugs Not Recommended by EMA or NICE Based on Clinical and Cost-Effectiveness Criteria **eTable 3.** Data and Indications for Figure Timeline
