# Supplementary material for: Assessment of Coverage in England of Cancer Drugs Qualifying for US Food and Drug Administration Accelerated Approval
Source: JAMA Intern Med. 2021 Feb 22;181(4):1–9. doi: 10.1001/jamainternmed.2020.8441 (PMC7900925; doi:10.1001/jamainternmed.2020.8441)
Supplement: Supplement. — eTable 1. Cancer Drug Indications With FDA Accelerated Approval Not Recommended or Not Reviewed by EMA or NICE eTable 2. Overview of Assessments for Cancer Drugs Not Recommended by EMA or NICE Based on Clinical and Cost-Effectiveness Criteria eTable 3. Data and Indications for Figure Timeline [file jamainternmed-e208441-s001.pdf]

Supplementary Online Content

Cherla A, Naci H, Kesselheim AS, Gyawali B, Mossialos E. Assessment of coverage in England of cancer drugs qualifying for US Food and Drug Administration accelerated approval. *JAMA Intern Med*. Published online February 22, 2021. doi:10.1001/jamainternmed.2020.8441

eTable 1. Cancer Drug Indications With FDA Accelerated Approval Not Recommended or Not Reviewed by EMA or NICE

eTable 2. Overview of Assessments for Cancer Drugs Not Recommended by EMA or NICE Based on Clinical and Cost-Effectiveness Criteria

eTable 3. Data and Indications for Figure Timeline

This supplementary material has been provided by the authors to give readers additional information about their work.

© 2021 Cherla A et al. *JAMA Internal Medicine*.

eTable 1. Cancer Drug Indications With FDA Accelerated Approval Not Recommended or Not Reviewed by EMA or NICE

Agency	Rejected or not reviewed	Drug	Reason
**EMA**	Not reviewed	Avelumab, durvalumab, everolimus x2, ibrutinib, irinotecan, pembrolizumab	Different indication from FDA.
		Belinostat, denileukin, everolimus tablets for oral suspension, omacetaxine, vinCRIStine liposome	Granted special rare disease designation and were not formally under review from the EMA by the end of our study period.
		Bicalutamide, oxaliplatin	Nationally authorized in EU countries, bypassing the EMA centralized review.
	Refused market authorization or withdrawn	Alemtuzumab, ofatumumab	Withdrawn by manufacturer for commercial reasons.
		Bevacizumab, pralatrexate, romidepsin	Refused marketing authorization for lacking evidence for safety or efficacy.
**NICE**	Not reviewed	Anastrozole, capecitabine, docetaxel, ibritumomab, imatinib x2, letrozole x2, lipocytarabine, liposomal doxorubicin, temozolomide	Available in the English market but not reviewed by NICE (no technology appraisal). Categorized as not routinely available. [a]
		Clofarabine, idelalisib, nelarabine, pembrolizumab, rucaparib	NICE review currently undergoing or suspended.
	Not recommended	Cetuximab, lapatinib, panitumumab, pemetrexed	Lacking evidence for clinical and cost-effectiveness.
		Carfilzomib, cetuximab, sunitinib	Lacking evidence for cost-effectiveness.

Abbreviations: FDA, US Food and Drug Administration; EMA; European Medicines Agency, EU; European Union, NICE; the National Institute for Health and Care Excellence.

[a] Not all drugs need to be recommended by NICE to be available in England's National Health Service (NHS). However, drugs without a positive recommendation from NICE do not have a funding mandate; therefore, they are not routinely available in the NHS. Their availability is subject to funding decisions from the relevant commissioning authority; NHS England or the local clinical commissioning group (bodies responsible for planning and commissioning medical services in a geographic area). Therefore, we categorized drug-indication pairs without a public technology appraisal as "not routinely available in the NHS."

© 2021 Cherla A et al. *JAMA Internal Medicine*.

eTable 2. Overview of Assessments for Cancer Drugs Not Recommended by EMA or NICE Based on Clinical and Cost-Effectiveness Criteria

Drug	Indication	FDA	EMA	NICE
Alemtuzumab	CLL	**Authorized**<br>Approved based on RR. Converted to regular approval (September 2007).<br>Reviewed: 7 May 2001	**Withdrawn**<br>Marketing authorization withdrawn at the request of the manufacturer (Genzyme) due to commercial reasons. Patients can still receive treatment through patient access programs.<br>Reviewed: 6 July 2001	**Not Reviewed**<br>Reviewed: N/A
Bevacizumab	Breast	**Withdrawn**<br>Confirmatory trial failed to verify clinical benefit; FDA commissioner issued withdrawal.<br>Reviewed: 22 December 2008 (approved)<br>18 November 2011 (withdrawn)	**Authorized**<br>Approved based on PFS. Benefit outweighs risk, however, use with docetaxel is not authorized. Also not authorized in combination with capecitabine for patients who have received anthracycline-containing regimens in the adjuvant setting within the last 12 months.<br>Reviewed: 23 July 2009	**Not Recommended**<br>**Reason**: Clinical and cost-effectiveness.<br>Small gain in PFS with no evidence for improved QoL. Cost per QALY greater than £82K.<br>Reviewed: 22 August 2012
Bevacizumab	Glioblastoma	**Authorized**<br>Approved based on RR. No overall survival improvement in post-market confirmatory trial. Converted to regular approval (December 2017).<br>Reviewed: 5 May 2009	**Refused Market Authorization**<br>No improvement in overall survival, PFS improved but with limited certainty due to clinical trial methods (limitation in the method used to measure brain tumor size).<br>Reviewed: 22 May 2014	**In development: suspended**<br>**Reason:** The EMA's CHMP (Committee for Medicinal Products for Human Use) adopted a negative review for the extension of the indication. This indication of bevacizumab was referred for further monitoring.<br>Reviewed: N/A
Cetuximab	mCRC	**Authorized**<br>Approved based on RR. Converted to regular approval (July 2017).<br>Reviewed: 12 February 2004	**Authorized**<br>Overall survival did not reach statistical significance in the randomized controlled trial (immature). Marginal improvement in PFS, statistically significant improvement in oRR.<br>Reviewed: 29 June 2004	**Not Recommended**<br>**Reason**: Clinical and cost-effectiveness.<br>Incremental difference in RR (primary endpoint) and overall survival was not statistically significant in the randomized controlled trial (immature). High level of uncertainty as clinical effectiveness could not

© 2021 Cherla A et al. *JAMA Internal Medicine*.

				be determined against the standard of care. Cost per QALY values of £77K – £370K.<br><br>Reviewed: 24 January 2007
Fludarabine	CLL	**Withdrawn**<br><br>Voluntarily withdrawn by Pfizer.<br><br>Reviewed: 18 December 2008 (approved)<br><br>31 December 2011 (withdrawn)	**Not Reviewed**<br><br>Nationally authorized in Belgium, not reviewed by EMA.<br><br>Reviewed: N/A	**Not Reviewed**<br><br>Not reviewed for this branded version (Oforta).<br><br>Reviewed: N/A
Gefitinib	NSCLC	**Withdrawn**<br><br>Confirmatory trial failed to verify clinical benefit, voluntarily withdrawn by the manufacturer (AstraZeneca). Subsequently approved in July 2015 as a first line treatment.<br><br>Reviewed: 5 May 2003 (approved)<br><br>25 April 2012 (withdrawn)	**Withdrawn**<br><br>Approved for patients after prior platinum-based chemotherapy but the submission was withdrawn by the manufacturer (AstraZeneca) in January 2005 as the overall survival data did not meet the requirements of the CHMP. A revised indication for previously untreated patients was resubmitted and subsequently authorized.<br><br>Reviewed: 1 January 2005	**Not Recommended**<br><br>**Reason**: Clinical and cost-effectiveness.<br><br>Absence of robust clinical evidence for PFS and overall survival, and without a proposed patient access scheme. Not possible to assess cost-effectiveness.<br><br>Reviewed: 16 December 2015
Gemtuzumab	AML	**Withdrawn**<br><br>Post-market confirmatory trial failed to verify benefit and indicated safety concerns. A revised submission with different indication, patient subgroup and lower dosage was accepted in September 2017.<br><br>Reviewed: 17 May 2000 (approved)<br><br>28 November 2011 (withdrawn)	**Refused Market Authorization**<br><br>Study design failed to demonstrate benefit and was insufficient to compare effectiveness without a comparator. Few patients achieved complete remission. Severe side effects; bone-marrow suppression, fever, chills. Resubmission approved in April 2018.<br><br>Reviewed: 20 September 2007	**Not Reviewed**<br><br>**Reason:** Similar to the FDA and EMA, gemtuzumab was accepted by NICE following resubmission for the revised indication in November 2018.<br><br>Reviewed: N/A
Lapatinib	Breast	**Authorized**<br><br>Approved based on PFS. Converted to regular approval (December 2018).	**Authorized**<br><br>Conditional approval based on PFS (primary endpoint). Overall survival evidence was	**Not Recommended**<br><br>**Reason**: Clinical and cost-effectiveness.<br><br>Improvement in PFS (primary endpoint), overall survival benefits small and uncertain

© 2021 Cherla A et al. *JAMA Internal Medicine*.

		Reviewed: 29 January 2010	immature, and the benefit was not statistically significant.<br><br>Reviewed: 5 May 2010	and no difference in QoL. Greater chance of adverse events. Cost per QALY of £74K.<br><br>Reviewed: 27 June 2012
Ofatumumab	CLL	**Authorized**<br><br>Approved based on RR. Converted to regular approval (April 2014)<br><br>Reviewed: 26 October 2009	**Withdrawn**<br><br>Marketing authorization withdrawn at the request of the manufacturer (Novartis) due to commercial reasons. Withdrawn outside the US only. Available outside the US for compassionate use programs and patients who still benefit.<br><br>Reviewed: 19 April 2010	**Withdrawn**<br><br>**Reason:** Marketing authorization withdrawn at the request of the manufacturer (Novartis) due to commercial reasons. Withdrawn outside the US only. Available outside the US for compassionate use programs and patients who still benefit.<br><br>Reviewed: N/A
Olaratumab	Sarcoma	**Withdrawn**<br><br>Failure to meet overall survival in the ANNOUNCE trial.<br><br>Reviewed: 19 October 2016 (approved)<br><br>24 January 2019 (withdrawn)	**Withdrawn**<br><br>ANNOUNCE trial indicated no improvement in overall survival or PFS more than doxorubicin alone.<br><br>Reviewed: 26 April 2019	**Withdrawn**<br><br>**Reason:** Lack of efficacy (June 2019).<br><br>Reviewed: N/A
Panitumumab	mCRC	**Authorized**<br><br>Approved based on PFS. Converted to regular approval (May 2014).<br><br>Reviewed: 27 September 2006	**Authorized**<br><br>Conditional approval based on improvement in PFS (primary endpoint), no significant improvement in overall survival (immature).<br><br>Reviewed: 3 December 2007	**Not Recommended**<br><br>**Reason:** Clinical and cost-effectiveness. PFS benefit of five weeks compared with best supportive care. Not a statistically significant improvement in overall survival. Cost per QALY values of £110K – £150K.<br><br>Reviewed: 25 January 2012
Pemetrexed	NSCLC	**Authorized**<br><br>Approved based on RR. Converted to regular approval (July 2009).<br><br>Reviewed: 12 August 2004	**Authorized**<br><br>Immature overall survival results not statistically significant based on the data from the non-inferiority trial. The EMA declared pemetrexed was as effective as its comparators.<br><br>Reviewed: 20 September 2004	**Not Recommended**<br><br>**Reason:** Clinical and cost-effectiveness.<br><br>No improvement in overall survival. Non-inferiority test did not remove the possibility of a marginal loss of efficacy when compared with docetaxel. Not cost-effective compared with docetaxel (> £1m per QALY) or best supportive care (> £50K per QALY).

© 2021 Cherla A et al. *JAMA Internal Medicine*.

				Reviewed: 22 August 2007
Pralatrexate	PTCL	**Authorized**<br><br>Approved based on RR.<br><br>Reviewed: 24 September 2009	**Refused Market Authorization**<br><br>Tumor response is not a clinical benefit endpoint and cannot be considered as a surrogate. Lacking evidence for efficacy of overall survival or PFS.<br><br>Reviewed: 19 January 2012	**In development: suspended**<br><br>**Reason:** Negative CHMP opinion. Referred for further monitoring.<br><br>Reviewed: N/A
Romidepsin	PTCL	**Authorized**<br><br>Approved based on RR.<br><br>Reviewed: 16 June 2011	**Refused Market Authorization**<br><br>Study did not include a comparator and was not possible to assess overall survival or PFS. Manufacturer failed to provide a certificate of Good Manufacturing Practice.<br><br>Reviewed: 12 February 2013	**In development: suspended**<br><br>**Reason:** CHMP adopted a negative review for extension of indication.<br><br>Reviewed: N/A
Sunitinib	RCC	**Authorized**<br><br>Approved based on RR. Converted to regular approval (Feb 2007).<br><br>Reviewed: 26 January 2006	**Authorized**<br><br>Conditional approval based on PFS.<br><br>Reviewed: 19 July 2006	**Not Recommended**<br><br>**Reason:** Clinical and cost-effectiveness.<br><br>Improvement in PFS, however an absence of robust data. Cost per QALY values of £72K and £105K.<br><br>Reviewed: 25 March 2009
Tositumomab	Lymphoma	**Withdrawn**<br><br>Voluntarily withdrawn by Corixa and GlaxoSmithKline.<br><br>Reviewed: 22 December 2004 (approved)<br><br>22 October 2013 (withdrawn)	**Not Reviewed**<br><br>Reviewed: N/A	**Not Reviewed**<br><br>Reviewed: N/A

Abbreviations: FDA, United States Food and Drug Administration; EMA, European Medicines Agency; NICE, the National Institute for Health and Care Excellence; MM, multiple myeloma; TTP, time-to-progression; CLL, chronic lymphocytic leukemia; US, United States; RR, response rate; PFS, progression-free survival; NSCLC, non-small cell lung cancer; oRR, objective response rate; QALY, quality adjusted life year; QoL, quality of life; mCRC, metastatic colorectal cancer; RCC, renal cell carcinoma; PTCL, peripheral t-cell lymphoma; CHMP, Committee for Medicinal Products for Human Use; AML, acute myeloid leukemia; OS, overall survival.

© 2021 Cherla A et al. *JAMA Internal Medicine*.

eTable 3. Data and Indications for Figure Timeline

Drug	Indication	FDA AA	FDA postmarket OS evidence	Withdrawn by FDA	EMA surrogate evidence	EMA OS evidence	EMA negative opinion	NICE surrogate evidence	NICE OS evidence	NICE negative opinion
Bicalutamide	Metastatic prostate cancer in combination with an LHRH analogue	10/4/1995								
Liposomal doxorubicin	AIDS-related Kaposi sarcoma after progression or intolerance to prior chemotherapy	11/17/1995			6/20/1996					
Docetaxel	Advanced or metastatic breast cancer after prior chemotherapy	5/14/1996	6/22/1998			9/19/2002				
Irinotecan	Metastatic colon or rectal cancer that has progressed after fluorouracil–based therapy	6/14/1996	10/22/1998							
Capecitabine	Metastatic breast cancer that is refractory to paclitaxel and to an anthracycline-containing regimen	4/30/1998	9/7/2001		10/18/2001					
Denileukin	Recurrent or persistent CTCL that expresses the CD25 component of the IL-2 receptor	2/5/1999								
Lipocytarabine	Intrathecal treatment for lymphomatous meningitis	4/1/1999			7/11/2001					

© 2021 Cherla A et al. *JAMA Internal Medicine*.

Liposomal doxorubicin	Metastatic ovarian cancer that is refractory to paclitaxel- and platinum-based regimens	6/28/1999			10/24/2000			7/1/2002		
Temozolomide	Refractory anaplastic astrocytoma after progression on a regimen that contains a nitrosourea and procarbazine	8/11/1999	3/15/2005		1/26/1999					
Gemtuzumab	CD33-positive AML in first relapse in patients ≥60 y of age who are not candidates for cytotoxic chemotherapy	5/17/2000		11/28/2011			9/20/2007			
Alemtuzumab	B-cell CLL that has been treated with alkylating agents and fludarabine	5/7/2001					7/6/2001			
Imatinib	CML in BC, AP, or CP after failure of interferon alfa therapy	5/10/2001			11/7/2001			10/22/2003		
Imatinib	Kit (CD117)–positive unresectable and/or metastatic malignant GIST	2/1/2002			5/24/2002			10/27/2004		
Ibritumomab	As part of a regimen for relapsed or refractory low-grade, follicular, or transformed B-cell NHL	2/19/2002			1/16/2004					
Oxaliplatin	In combination with fluorouracil and leucovorin for mCRC that has recurred or progressed with fluorouracil and	8/9/2002	1/9/2004							

© 2021 Cherla A et al. *JAMA Internal Medicine*.

	leucovorin plus irinotecan									
Anastrazole	Adjuvant, postmenopausal, HR-positive early breast cancer	9/5/2002								
Imatinib	Newly diagnosed, Ph-positive CML	12/20/2002			11/7/2001			10/22/2003		
Gefitinib	Monotherapy for locally advanced or metastatic NSCLC after platinum-based and docetaxel therapies	5/5/2003		4/25/2012			1/1/2005			12/16/2015
Bortezomib	MM with at least 2 prior therapies	5/13/2003	3/25/2005		4/26/2004				10/23/2007	
Imatinib	Pediatric, Ph-positive CP CML resistant to interferon or recurrent after SCT	5/20/2003								
Cetuximab	Single agent for EGFR-positive mCRC intolerant to irinotecan-based chemotherapy	2/12/2004	10/2/2007		6/29/2004					1/25/2012
Cetuximab	With irinotecan in EGFR-positive mCRC refractory to irinotecan-based chemotherapy	2/12/2004			6/29/2004					1/24/2007
Pemetrexed	Locally advanced or metastatic NSCLC after previous chemotherapy	8/19/2004	7/2/2009		9/20/2004					8/22/2007
Letrozole	Extended adjuvant postmenopausal breast	10/29/2004								

© 2021 Cherla A et al. *JAMA Internal Medicine*.

	cancer after 5 years of tamoxifen therapy									
Tositumomab	Relapsed or refractory low-grade follicular not treated with rituximab	12/22/2004		10/23/2013						
Clofarabine	Pediatric relapsed/ refractory ALL	12/28/2004			5/29/2006					
Nelarabine	Relapsed/refractory Tcell ALL or T-cell lymphoblastic lymphoma	10/28/2005			8/22/2007					
Letrozole	Adjuvant, post-menopausal, HR-positive breast cancer	12/28/2005								
Sunitinib	Advanced RCC	1/26/2006			7/19/2006					3/25/2009
Thalidomide	Newly diagnosed MM	5/25/2006				4/16/2008			7/27/2011	
Dasatinib	CML that is resistant or intolerant to prior therapy, including imatinib	6/28/2006			11/20/2006			1/1/2012		
Panitumumab	EGFR expressing mCRC after fluoropyrimidine-, oxaliplatin-, and irinotecan-containing regimens	9/27/2006	5/23/2014		12/3/2007					1/25/2012
Imatinib	Newly diagnosed, pediatric, Ph-positive CML	9/27/2006			3/19/2009					
Nilotinib	Ph-positive CML CP or AP resistant or intolerant to imatinib	10/29/2007			11/19/2007			12/21/2016		

© 2021 Cherla A et al. *JAMA Internal Medicine*.

Pemetrexed	Locally advanced or metastatic NSCLC with cisplatin	9/26/2008	7/2/2009		4/8/2008				9/23/2009	
Imatinib	Adjuvant treatment after complete gross resection of Kit (CD117)–positive GIST	12/19/2008	1/31/2012		4/29/2009			11/26/2014		
Fludarabine	B-cell CLL after at least 1 standard alkylating agent–containing regimen	12/18/2008		12/31/2011						
Bevacizumab	First line in combination with paclitaxel for metastatic, HER2-negative breast cancer	12/22/2008		11/18/2011	7/23/2009					8/22/2012
Bevacizumab	Glioblastoma after progression on chemotherapy/radiation therapy	5/5/2009					5/22/2014			
Pralatrexate	Refractory/relapsed PTCL	9/24/2009					1/19/2012			
Ofatumumab	Chronic lymphocytic leukaemia refractory to fludarabine and alemtuzumab	10/26/2009					4/19/2010			
Lapatinib	In combination with letrozole in postmenopausal women with hormone receptor+ and HER2+ metastatic breast cancer for whom hormonal therapy is indicated	1/29/2010			5/5/2010					6/27/2012
Nilotinib	Newly diagnosed, Ph-positive CML in CP	6/17/2010			12/20/2010			12/21/2016		

© 2021 Cherla A et al. *JAMA Internal Medicine*.

Dasatinib	Newly diagnosed, Ph-positive CML in CP	10/28/2010			12/6/2010			12/21/2016		
Everolimus	SEGA associated with TSC that is not a resection candidate	10/29/2010								
Romidepsin	PTCL after at least one prior therapy	6/16/2011					2/12/2013			
Brentuximab vedotin	HL after failure of ASCT or at least 2 prior multiagent chemo regimens in patients not ASCT candidates	8/19/2011			10/25/2012			6/13/2018		
Brentuximab vedotin	sALCL after failure of at least one prior multiagent chemotherapy regimen	8/19/2011			10/25/2012			10/4/2017		
Crizotinib	Locally advanced or metastatic ALK mutation–positive NSCLC	8/26/2011			10/23/2012			9/28/2016		
Everolimus	Renal angiomyolipoma associated with TSC who do not require immediate surgery	4/26/2012								
Carfilzomib	MM after at least 2 prior therapies, including bortezomib and an immunomodulatory agent	6/20/2012				11/19/2015				7/19/2017
vincristine liposome	Philadelphia chromosome-negative ALL in 2nd or greater relapse or that has progressed following 2	8/9/2012								

© 2021 Cherla A et al. *JAMA Internal Medicine*.

	or more anti-leukemia therapies									
Everolimus tablets for oral suspension	Pediatric and adult patients with TSC who have SEGA that requires therapeutic intervention but cannot be curatively resected	8/29/2012								
Omacetaxine	Chronic or accelerated CML after resistance or intolerance to 2 or more TKIs	10/26/2012								
Ponatinib	CP, AP, or BC CML resistant or intolerant to prior TKI or Ph-positive ALL resistant or intolerant to prior TKIs	12/14/2012			7/1/2013			6/28/2017		
Pomalidomide	MM after at least 2 prior therapies, including lenalidomide and bortezomib	2/8/2013			8/5/2013			1/11/2017		
Pertuzumab	Neoadjuvant HER2+ breast cancer	9/13/2013			7/28/2015			12/21/2016		
Ibrutinib	Mantle Cell Lymphoma	11/13/2013			10/21/2014			January 31 2018		
Trametinib	In combination with dabrafenib for the treatment of patients with unresectable or metastatic melanoma with BRAF V600E or V600K mutations	1/8/2014	11/20/2015		6/30/2014			10/17/2018		

© 2021 Cherla A et al. *JAMA Internal Medicine*.

Dabrafenib	In combination with trametinib for the treatment of patients with unresectable or metastatic melanoma with BRAF V600E or V600K mutations	1/9/2014	11/20/2015		8/25/2015			10/17/2018		
Ibrutinib	Treatment of CLL after 1 prior therapy	2/12/2014	7/28/2014		10/21/2014			1/25/2017		
Ceritinib	Treatment of ALK mutation–positive metastatic NSCLC after progression or intolerance to crizotinib	4/29/2014			5/6/2015			6/22/2016		
Belinostat	Relapsed or refractory PTCL	7/3/2014								
Idelalisib	Relapsed follicular Bcell NHL or relapsed SLL after at least 2 prior therapies	7/23/2014			10/14/2014					
Pembrolizumab	Unresectable or metastatic melanoma after ipilimumab and a BRAF inhibitor if BRAF mutation positive	9/4/2014			7/17/2015			10/7/2015		
Blinatumomab	Philadelphia chromosome-negative relapsed or refractory Bcell precursor acute lymphoblastic leukemia	12/3/2014	12/29/2015		11/23/2015				6/28/2017	
Olaparib	Monotherapy for gBRCA ovarian cancer after 3 or more prior lines of chemotherapy	12/19/2014			12/16/2014			1/27/2016		

© 2021 Cherla A et al. *JAMA Internal Medicine*.

Nivolumab	Unresectable/metastatic melanoma and progression following ipilimumab and, if BRAF mutation positive, a BRAF inhibitor	12/22/2014			6/19/2015			2/18/2016		
Palbociclib	In combination with letrozole for postmenopausal, ER-positive HER2 metastatic breast cancer as initial endocrine therapy	2/3/2015			11/9/2016			12/20/2017		
Panobinostat	In combination with bortezomib and dexamethasone for MM after at least 2 prior regimens including bortezomib and an immunomodulatory agent	2/23/2015			8/28/2015			1/27/2016		
Nivolumab	In combination with ipilimumab for BRAF wild type metastatic melanoma	9/30/2015	8/1/2016		5/11/2016			7/27/2016		
Pembrolizumab	PDL1-positive metastatic NSCLC after platinum-containing chemotherapy	10/2/2015	10/24/2016			6/23/2016			1/11/2017	
Osimertinib	Metastatic EGFR T790M mutation–positive NSCLC after progression on or after an EGFR TKI	11/13/2015			2/1/2016			10/26/2016		

© 2021 Cherla A et al. *JAMA Internal Medicine*.

Daratumumab	MM after at least 3 prior lines of therapy, including a proteasome inhibitor and an immunomodulatory drug or are double refractory	11/16/2015			4/28/2017			3/14/2018		
Alectinib	ALK mutation positive NSCLC progressing on crizotinib	12/11/2015			2/16/2017			8/8/2018		
Venetoclax	CLL with 17p deletion after one prior therapy	4/11/2016			12/4/2016			11/8/2017		
Nivolumab	cHL after autologous SCT and brentuximab vedotin	5/17/2016			10/13/2016			7/26/2017		
Atezolizumab	Locally advanced or metastatic urothelial carcinoma after platinum therapy	5/18/2016			9/20/2017			6/13/2018		
Pembrolizumab	Squamous cell carcinoma of the head and neck on or after progression with platinum	8/5/2016	5/15/2017			9/4/2018				
Olaratumab	With Doxil for soft tissue sarcoma	10/19/2016		1/24/2019			4/26/2019			
Rucaparib	Monotherapy for the treatment of patients with deleterious BRCA mutation ovarian cancer after 2 or more lines of chemotherapy	12/19/2016			5/23/2018					
Ibrutinib	Marginal Zone Lymphoma requiring systemic therapy & have previously received at	1/18/2017								

© 2021 Cherla A et al. *JAMA Internal Medicine*.

	least one prior anti-CD20-based therapy									
Nivolumab	Locally advanced or metastatic urothelial carcinoma after platinum therapy	2/2/2017			6/2/2017			7/4/2018		
Pembrolizuma b	Refractory cHL or those who have relapsed after 3 or more prior lines of therapy	3/14/2017			5/2/2017			9/3/2018		
Avelumab	Metastatic Merkel Cell Carcinoma	3/23/2017			9/18/2017			4/11/2018		
Atezolizumab	Locally advanced or metastatic urothelial carcinoma not eligible for cisplatin-containing chemotherapy	4/17/2017			9/20/2017			6/12/2017		
Nivolumab	cHL that has relapsed/progressed after autologous SCT and brentuximab vedotin or after 3 or more lines that includes autologous SCT	4/25/2017			10/13/201 6			7/26/2017		
Brigatinib	Patients with ALK mutation-positive metastatic NSCLC who have progressed on or are intolerant to crizotinib	4/28/2017			11/22/201 8			3/20/2019		
Durvalumab	Locally advanced or metastatic urothelial carcinoma after platinum therapy	5/1/2017								

© 2021 Cherla A et al. *JAMA Internal Medicine*.

Avelumab	Locally advanced or metastatic urothelial carcinoma after platinum therapy	5/9/2017								
Pembrolizumab	First-line treatment in patients with nonsquamous NSCLC in combination with pemetrexed and carboplatin	5/10/2017	11/8/2017		9/4/2018			1/10/2019		
Pembrolizumab	Locally advanced or metastatic urothelial carcinoma not eligible for cisplatin-containing chemotherapy	5/18/2017			7/6/2018			6/13/2018		
Pembrolizumab	Unresectable/metastatic MSI-H/dMMR solid tumors that have progressed on prior treatment or that have no alternative options, or, MSI-H/dMMR mCRC that has progressed on 5FU, oxaliplatin, and irinotecan	5/23/2017								

Abbreviations: FDA, United States Food and Drug Administration; EMA, European Medicines Agency; NICE, the National Institute for Health and Care Excellence; AA, accelerated approval; OS, overall survival.

© 2021 Cherla A et al. *JAMA Internal Medicine*.
